# Star‐Shaped Boron‐Containing Asymmetric Host Materials for Solution‐Processable Phosphorescent Organic Light‐Emitting Diodes

**DOI:** 10.1002/advs.201800292

**Published:** 2018-05-28

**Authors:** Jibiao Jin, Ye Tao, He Jiang, Runfeng Chen, Guohua Xie, Qin Xue, Chen Tao, Lu Jin, Chao Zheng, Wei Huang

**Affiliations:** ^1^ Key Laboratory for Organic Electronics and Information Displays & Jiangsu Key Laboratory for Biosensors Institute of Advanced Materials (IAM) Jiangsu National Synergistic Innovation Center for Advanced Materials (SICAM) Nanjing University of Posts & Telecommunications 9 Wenyuan Road Nanjing 210023 China; ^2^ Hubei Collaborative Innovation Center for Advanced Organic Chemical Materials Hubei Key Lab on Organic and Polymeric Optoelectronic Materials Department of Chemistry Wuhan University Wuhan 430072 China; ^3^ Department of Physical Science and Technology Central China Normal University Wuhan 430079 China; ^4^ School of Physics and Technology Wuhan University Wuhan 430072 China

**Keywords:** boron‐containing materials, host materials, organic light‐emitting diodes, solution processing

## Abstract

Boron‐containing compounds have attracted considerable attention because of their electron‐accepting properties, and they are widely used in a variety of fields. However, due to the essential requirement to protect the empty p_z_‐orbital of the boron atom using large steric hindrance or rigid groups, borane derivatives generally show poor solubility and are rarely reported as acceptor units to construct bipolar host materials for phosphorescent organic light‐emitting diodes (PhOLEDs). Here, a combined star‐shaped and asymmetric donor–acceptor molecular design strategy to improve the solubility and fine tune the optical and electronic properties of boron‐containing materials is presented. High thermal stability, solvent solubility, solution processability, and triplet energy are achieved simultaneously. With the thus‐designed boron‐containing bipolar molecules as host materials, the solution‐processed PhOLEDs exhibit high device performances, which are comparable to the vacuum‐processed counterparts, showing high external quantum efficiencies up to 18.5% and 14.5% in blue and white PhOLEDs, respectively. These results demonstrate the great potential of the star‐shaped and symmetry‐breaking borane derivatives in solution‐processable organic optoelectronic devices.

## 1 Introduction

Boron, a versatile element with an empty p_z_‐orbital, tends to form electron‐deficient multiatom networks in three‐coordination and trigonal‐planar geometry, arousing fascinating characteristics when being introduced into the organic optoelectronic molecules.^1^, ^2^ Boron‐containing molecules with strong electron‐accepting triarylboron moiety have received considerable attention, because they are potential materials for a number of photonic and optoelectronic applications, such as nonlinear optic molecules,^3^ anion sensing probes,^4^ electron‐transporting materials,^5^, ^6^ fluorescent dyes,^7^ and thermally activated delayed fluorescent compounds.^8^, ^9^, ^10^ However, borane derivatives are rarely used as electron‐accepting species to construct bipolar host materials for phosphorescent organic light‐emitting diodes (PhOLEDs), probably due to the high activity of the empty boron p_z_‐orbital in binding any Lewis bases, although almost all the other requirements of host materials can be fulfilled:^11^, ^12^, ^13^ 1) high triplet energy (*E*_T_) to facilitate exothermic energy transfer from host to guest molecules and confine triplet excitons on guest molecules for emission, 2) bipolar features with high carrier mobility and suitable the highest occupied molecular orbital (HOMO), and the lowest unoccupied molecular orbital (LUMO) energy levels to support balanced and efficient carrier injection and transportation during the operation of PhOLEDs, and 3) high thermal stability and good miscibility with the guest molecules for stable amorphous and uniformly doped films through vacuum thermal evaporation and upon heating.

To prepare stable triarylboranes that free of chemical attack, steric hindrance around the boron center has been recognized as a useful way to protect the empty p_z_‐orbital of boron atom in constructing applicable optoelectronic molecules.^2^ In 2012, by combining bulky dimesityl borane and rigid carbazole, Wu and co‐workers first reported the boron‐containing host materials for blue and white PhOLEDs, achieving the corresponding external quantum efficiencies (EQEs) of 16.5% and 15.7% in vacuum‐deposited devices.^14^ In 2016, Liao and co‐workers used also the bulky dimesityl borane as acceptor (A) but more rigid spiro[acridine‐9,9′‐fluorene] as donor (D) to design boron‐based bipolar host materials for blue PhOLEDs with high EQE up to 25.1%.^15^ Very recently, they linked the dimesityl borane to the N‐atom of bicarbazole and thus obtained boron‐containing host material showed a maximum EQE of 16.7% in blue PhOLEDs.^16^ In spite of these high device performances reported recently, the development of boron‐based host materials lags far behind in comparison to that of the other types of host molecules. Even more tragically, the bulky steric hindrance and rigid groups employed to kinetically prevent the attack of nucleophiles generally lead to the poor solubility of the boron‐containing compounds in solvents; this inevitably prohibits their applications in solution‐processed devices, although the solution processing in fabricating devices has widely recognized advantages than the vacuum thermal deposition technology and is in great demand for low‐cost and large‐area production of optoelectronic devices in commercial manufacture.^11^, ^17^, ^18^

**Scheme 1:**
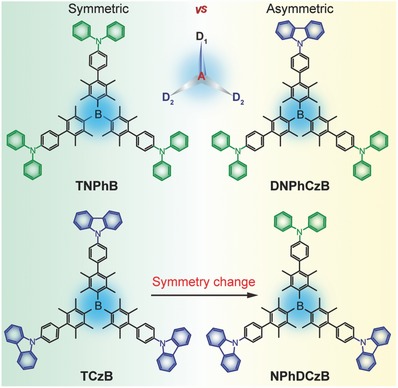
Molecular design and chemical structures of the symmetric (left) and asymmetric (right) star‐shaped boron‐containing D–A molecules of **TNPhB**, **DNPhCzB**, **NPhDCzB**, and **TCzB**.

Here, we aim to address this intrinsic obstacle by combining the star‐shaped and asymmetric molecular design strategies together to improve the solubility and fine tune the optical and electronic properties of the boron‐containing D–A molecules with an acceptor core of bulky tetramethylphenyl borane and varied peripheral donors of carbazole and diphenylamine (**Scheme** **1**). It is well known that star‐shaped molecules are promising candidates for solution‐processable optoelectronic devices;^19^ meanwhile, the asymmetric materials could finely modulate the photophysics and electrochemistry properties by increasing the number of the conformers and amount of energy for crystallization to favor the stability improvement of amorphous film.^20^ Since carbazole is a weak donor and diphenylamine is a strong one, the competition and cooperation of the different number and types of donors in the star‐shaped symmetry‐breaking D–A molecular architecture would significantly modify the molecular structures and optoelectronic properties of asymmetric molecules. Indeed, good solubility and high stability were observed in these asymmetric star‐shaped boron‐containing D–A molecules; high *E*_T_ and suitable HOMO and LUMO qualify them as solution‐processable bipolar host materials for blue PhOLEDs. Impressively, the solution‐processed PhOLEDs using asymmetric host materials of **DNPhCzB** and **NPhDCzB** exhibit comparable device performances to those of vacuum‐fabricated devices, showing low turn‐on voltage of 3.5 V, high EQEs of 18.4% and 18.5% for blue PhOLEDs, and of 10.7% and 14.5% for white PhOLEDs in simplified device structures.

## 2 Result and Discussion

### 2.1 Molecular Design and Synthesis

The boron‐containing star‐shaped D–A molecules of **TNPhB**, **DNPhCzB**, **NPhDCzB**, and **TCzB** (Scheme 1) were designed to have the same electron‐withdrawing core of triarylborane with tetramethylphenyl propeller arrangements, which effectively shields the boron p_z_‐orbital from attack by Lewis bases or nucleophiles and facilitates the forming of amorphous films through solution‐processing. Then, carbazole or diphenylamine, which has been extensively applied in PhOLEDs with high *E*_T_ and hole‐transport properties,^21^ were introduced symmetrically or asymmetrically as the electron‐donating moiety to the three branches of the triarylborane core. Based on the facilely prepared tris‐brominated triarylborane core, these compounds in white powders were efficiently synthesized in high yields (72–88%) through one or two steps of classical Suzuki coupling reactions (Scheme S1, Supporting Information).^22^ Detailed synthetic route and systematic characterizations through ^1^H/^13^C NMR spectroscopy and high resolution mass spectrum are given in Figures S1–S4 in the Supporting Information. Owing to the star‐shaped molecular architecture, all the compounds exhibit good solubility (>30 mg mL^−1^) in common organic solvents (Table S1, Supporting Information), and these asymmetric molecules of **DNPhCzB** and **NPhDCzB** demonstrate even higher solubility (>150 mg mL^−1^) than the symmetric compounds due to the effects of asymmetric architectures, which could increase the number of the conformers with weaker intermolecular interaction.

### 2.2 Thermophysical and Photophysical Properties

Differential scanning calorimetry (DSC) and thermal gravimetric analysis (TGA) reveal that these boron‐containing compounds have good thermal stability with high decomposition temperatures (*T*_d_ > 416 °C), glass‐transition temperatures (*T*_g_ > 158 °C), and melting temperature (*T*_m_ > 213 °C) owing to the stable triarylborane core with triple protection of tetramethylphenyl and peripheral aromatic carbazole and diphenylamine branches (Figure S5, Supporting Information). Impressively, higher *T*_g_, *T*_m_, and *T*_d_ were observed in asymmetric molecules of **DNPhCzB** and **NPhDCzB** than in **TNPhB** and **TCzB**; and, all these values are high enough for the host material applications of OLEDs.^17^ The enhancement effects of asymmetric molecular architecture were also observed on the film morphology, showing smooth surface with slightly smaller root‐mean‐square roughness (RMS) of the spin‐coated amorphous thin films (Figure S6, Supporting Information). Therefore, the better film morphology and higher solubility of the asymmetric boron‐containing molecules with improved thermal stability suggest their promising potential in the fabrication of organic optoelectronic devices through solution‐processing.

**Figure 1:**
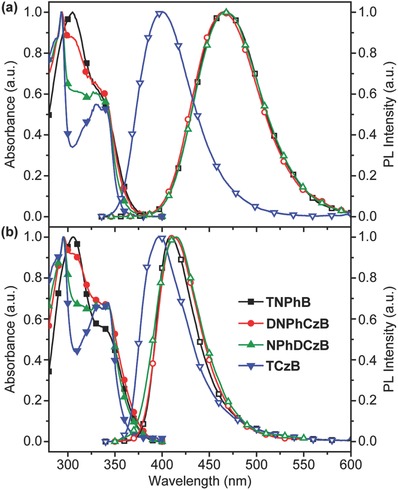
UV‐absorption (solid symbols) and photoluminescence (PL) spectra (open symbols) of **TNPhB**, **DNPhCzB**, **NPhDCzB**, **TCzB** in a) CH_2_Cl_2_ solution (≈10^−5^ mol L^−1^) and b) solid films.

The photophysical properties of the star‐shaped boron‐containing compounds were investigated by UV–vis absorption and photoluminescence (PL) spectra (**Figure** **1** and **Table** **1**) in dilute CH_2_Cl_2_ solution (≈10^−5^ mol L^−1^) and thin solid film. In both solution and solid states, the absorption spectra of these boron‐containing compounds are very similar, exhibiting one distinct absorption band peaked at around 295 nm which can be ascribed to the carbazole or diphenylamine‐centered π–π* transition, and a weaker absorption at longer wavelength around 340 nm that should be due to the n–π* transitions of the N‐containing molecule.^23^ The weak absorption band is also very close to the absorption peak of the triarylborane core around 332 nm (Figure S7, Supporting Information). Moreover, it is very stable in different solvents with varied polarities (Figure S8, Supporting Information), excluding the possible absorption of intramolecular charge transfer (ICT) between the donor and acceptor. Therefore, the second absorption band could be resulted by both the n–π* transitions and the absorption of the triarylborane core.

**Table 1:** Photophysical and electrochemical properties of the boron‐containing D–A molecules

Compound	*T*_d_/*T*_g/m_ [°C]	λ_abs_ [nm]		^opt^ *E*_g_ c) [eV]	λ_em_ [nm]		*E*_T_ d) [eV]	CV [eV]	
		CH_2_Cl_2_	Film		CH_2_Cl_2_	Film		HOMO	LUMOe)
**TNPhB**	416/158a)	310, 338	302, 330	3.32	467	408	2.77	−5.34	−2.02
**DNPhCzB**	453/161a)	298, 334	295, 333	3.31	467	412	2.80	−5.35	−2.04
**NPhDCzB**	448/274b)	298, 334	295, 333	3.30	468	415	2.87	−5.40	−2.10
**TCzB**	416/213b)	298, 334	295, 333	3.45	403	396	2.90	−5.66	−2.21

^a)^ *T*_g_

^b)^ *T*_m_

^c)^ Optical bandgap (^opt^ *E*_g_) calculated by the absorption edge technique in solid film

^d)^ Triplet energy (*E*_T_) measured from phosphorescent spectrum at 77 K with a delay time of 5 ms

^e)^ LUMO energy level estimated by adding *E*_g_ to the HOMO energy level.

Upon photoexcitation at the absorption maxima at room temperature, broad and structureless emission bands appear with large Stokes shifts up to 134 nm in photoluminescence spectra of these boron‐containing compounds in CH_2_Cl_2_ solution, suggesting clearly the ICT emission characteristics due to the charge transfer from the donor of carbazole or diphenylamime to the acceptor of triarylborane core (Figure 1a).^10^ The lower electron‐donating ability of carbazole than that of diphenylamime leads to the lowest Stokes shift in **TCzB**, whereas the largest Stokes shifts was not found in **TNPhB** but in asymmetric **DNPhCzB** and **NPhDCzB**, indicating again the great impact of asymmetric molecular architecture. The ICT feature of their emission was further confirmed by the large variations of their luminescent peaks in solvents with different polarities (Figure S8, Supporting Information). Interestingly, contrary to the widely observed red‐shift of the emission band in solid state, these molecules show blue‐shifted photoluminescence spectra in thin films than in CH_2_Cl_2_ (Figure 1b). This could be due to the characteristic property of boron‐containing D–A molecules, exhibiting small dipole moments at ground states for almost unchanged absorption bands in different solvents and solid states but large dipole moments at excited states to response significantly to the molecular state change.^24^, ^25^

**Figure 2:**
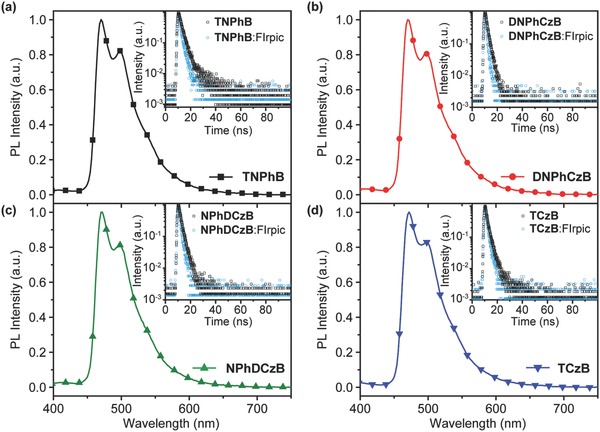
Photoluminescent (PL) spectra of FIrpic‐doped (5 wt%) a) **TNPhB,** b) **DNPhCzB,** c) **NPhDCzB**, and d) **TCzB** films and transient PL decay curves of the boron‐containing materials with or without 1 wt% FIrpic (inset) excited at 330 nm.

From the onset thresholds of the absorption spectra in solid films, the optical band gaps (^opt^ *E*_g_s) of **TNPhB**, **DNPhCzB**, **NPhDCzB**, and **TCzB** were identified to be as wide as 3.32, 3.31, 3.30, and 3.45 eV, respectively. From the time‐resolved phosphorescence spectra (Figure S9, Supporting Information) measured at 77 K in CH_2_Cl_2_, the lowest triplet excited states (T_1_) energy (*E*_T_s) of these molecules were evaluated to be as high as 2.77, 2.80, 2.87, and 2.90 eV for **TNPhB**, **DNPhCzB**, **NPhDCzB**, and **TCzB**, respectively. To our delight, the *E*_T_s of all these compounds are successfully designed to be higher than that of the popular blue phosphorescent emitter of bis[2‐(4,6‐difluorophenyl) pyridinato‐C^2^,N] (picolinato) iridium (III) (FIrpic).^26^ To experimentally confirm the efficient energy transfer from the boron‐containing host material to FIrpic, both steady‐state and time‐resolved photoluminescent investigations were performed (**Figure** **2**). Under the irradiation of 330 nm UV‐light to excite the host molecules, the composite films with 5 wt% doped FIrpic show only the characteristic emission bands of the dopant without the apparent luminescent peaks of the host materials. Moreover, the emission lifetimes of the host materials doped by 1 wt% FIrpic are reduced remarkably in comparison to that of the dopant‐free films, presenting direct evidence for the direct energy transfer from the host to the guest molecules. Thus, we would conclude that these star‐shaped boron‐containing compounds are potentially good host materials for blue PhOLEDs capable of providing effective confinement of the triplet excitons on the guest of FIrpic and preventing back energy transfer from the guest to the host molecules for high device performances.

### 2.3 Electrochemical and Computational Investigations

**Figure 3:**
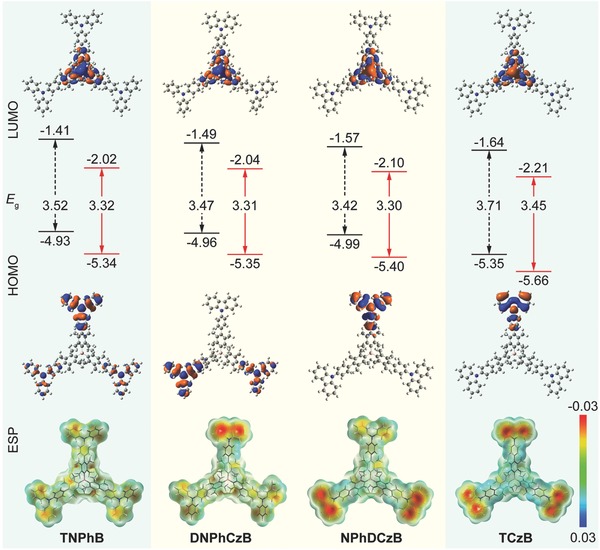
Theoretical (black) and experimental (red) energy levels of **TNPhB**, **DNPhCzB**, **NPhDCzB**, and **TCzB**, as well as the isosurfaces of the frontier orbitals and electrostatic potential (ESP). The potential energy range is −0.03–0.03 H q^−1^ for all surfaces shown, where red indicates areas with dense electron density, yellow for normal, and blue areas suggest less electron density.

The electrochemical properties of the star‐shaped boron‐containing D–A molecules were investigated by cyclic voltammetry (CV) using tetrabutylammonium hexafluorophosphate (TBAPF_6_) as the supporting electrolyte and ferrocene as the internal standard.^27^ From the onset of the oxidation wave, the HOMO energy levels of **TNPhB**, **DNPhCzB**, **NPhDCzB**, and **TCzB** were measured to be −5.34, −5.35, −5.40, and −5.66 eV, respectively (Figure S10, Supporting Information). With the aid of their optical band gaps, their LUMO energy levels were estimated to be −2.02, −2.04, −2.10, and −2.21 eV, respectively. These experimentally measured frontier orbital energy levels are well in line with those theoretically predicted by the density functional theorty (DFT) calculations, demonstrating the highest HOMO in **TNPhB** and lowest LUMO in **TCzB** due to the stronger electron‐donating property of diphenylamine than that of carbazole (**Figure** **3**). Impressively, the asymmetric molecules of **DNPhCzB** and **NPhDCzB** can inherit both the high HOMO from **TNPhB** and low LUMO from **TCzB**, resulting in the lowest bandgap in **NPhDCzB**. It should be also noted that these HOMO and LUMO energy levels are close to those of the widely used hole‐transporting and electron‐transporting materials, suggesting their good potentials in optoelectronic device applications.

Further, from the frontier orbital isosurfaces, the acceptor of triarylborane core dominates the LUMO, while the HOMO is determined mainly by the peripheral donors of diphenylamine or carbazole in these star‐shaped D–A molecules, demonstrating the separated frontier orbital distributions for the typical bipolar charateristic.^28^ When diphenylamine and carbazole coexist on the peripheral branches asymmetricly in **DNPhCzB** and **NPhDCzB**, HOMO tends to be dominated by diphenylamine due to its stronger electron‐donating ability, leading to their close HOMO energy levels to that of **TNPhB** (Figure 3). The interaction modes of carbazole and diphenylamine with the triarylborane core should be different, because higher electrostatic potential (ESP) was observed on carbazole moieties, although ESP distributions still delocalize on the whole molecular architectures due to the extension of π‐conjugation in these star‐shaped D–A molecules. The less negative ESP values on diphenylamine could be due to its better electronic communication between the triarylborane core to delocalize more efficiently its electrons on the acceptor. This efficient electron communication should be also an important reason for the much lower bandgaps of the diphenylamine‐containing molecules.

From the spin density distributions (Figure S11, Supporting Information), carbazole and diphenylamine tend to dominate the T_1_ state. In contrast, the T_1_ of **NPhDCzB** was codetermined by diphenylamine and triarylborane. Therefore, superior *E*_T_ of the triarylborane core, higher than that of carbazole and diphenylamine, can be inferred according to the fact that the lowest *E*_T_ moiety determines the T_1_ of the whole molecule. Such high *E*_T_ of the triarylborane core could be due to its nonconjugated central C–B bond with highly twisted bulky tetramethylphenyl propeller substituents, resulting in the limited conjugation length along the boron‐containing core. This observation suggests that our triarylborane core is highly attractive as high‐performance acceptor unit for the design of bipolar host materials with high *E*_T_ for blue PhOLEDs.

### 2.4 Solution‐Processed PhOLEDs Hosted by the Star‐Shaped Boron‐Containing D–A Molecules

**Figure 4:**
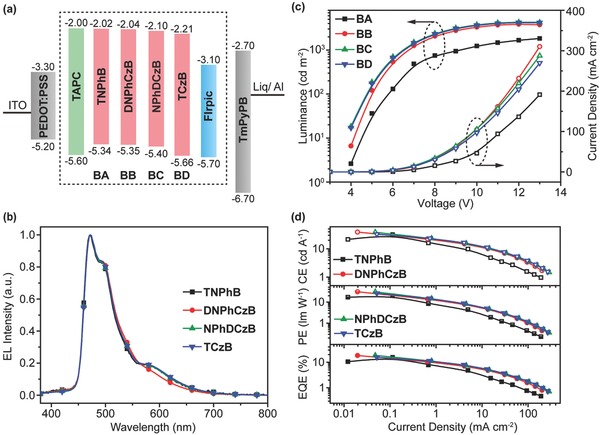
a) Device configuration and energy level diagram of the materials used in the spin‐coated blue PhOLEDs based on host materials of **TNPhB, DNPhCzB, NPhDCzB**, and **TCzB**. b) Electroluminescent spectra at 7.0 V. c) Current density–voltage (open symbols) and luminance–voltage (solid symbols) curves, d) Efficiency–current density curves.

In light of the high solubility, stability, and *E*_T_ as well as suitable frontier orbital energy levels of these star‐shaped boron‐containing D–A molecules, we used them as host materials for the FIrpic‐based blue PhOLEDs fabricated through solution‐processing under the simplified device configuration of indium tin oxide (ITO)/poly(3,4‐ethylenedioxythiophene):poly(styrenesulfonic acid) (PEDOT:PSS) (80 nm)/boron‐containing molecule: 1,1‐bis[(di‐4‐tolylamino)phenyl] cyclohexane (TAPC) (× wt%): 15 wt% FIrpic (35 nm)/TmPyPB (40 nm)/Liq (1 nm)/Al (100 nm) (**Figure** **4**a).^11^ In these devices, PEDOT:PSS and 8‐hydroxyquinolinolato‐lithium (Liq) serve as the hole‐ and electron‐injecting layer, respectively; FIrpic as the blue lighting phosphorescent guest, **TNPhB**, **DNPhCzB**, **NPhDCzB**, **TCzB,** and TAPC as the hosts, and 1,3,5‐tri[(3‐pyridyl)‐phen‐3‐yl]benzene (TmPyPB) as the hole‐blocking and electron‐transporting material. The incorporation of TAPC in the host material can, to some extent, improve the morphology of the spin‐coated film (Figure S6, Supporting Information) and increase the hole‐transport properties of the emitting layers.

Efficient exothermic energy transfer from the host to the guest molecules was verified by both the photoluminescent (Figure S12, Supporting Information) and electroluminescent spectra (Figure 4b; Figure S13, Supporting information) of the FIrpic‐doped emitting layer. Only the characteristic emission bands of FIrpic were observed with a small peak of TAPC‐induced excimer emission around 580 nm^29^ in all the host–guest composite films without any emissions of host molecules at different contents of TAPC (Figure S13, Supporting Information). Time‐resolved emission decay curves of FIrpic exhibit monoexponential decay behavior (Figure S14, Supporting Information), indicating the well confinement of excitons on FIrpic and efficient energy transfer from the host to guest.^30^ Furthermore, the short emission lifetime of FIrpic around 1.0 µs would be helpful to alleviate the triplet state involved quenching effects in the emitting layer; this is favorable for the high‐performance devices.^26^ Therefore, at the optimized TAPC content (Table S2, Supporting Information), the blue PhOLEDs hosted by the star‐shaped boron‐containing bipolar host materials reach the maximum current efficiencies (CE) of 31.8, 38.9, 38.5, and 32.8 cd A^−1^, power efficiencies (PE) of 20.0, 30.5, 30.2, and 25.8 lm W^−1^, and EQEs of 15.6%, 18.4%, 18.5%, and 15.8% at turn‐on voltages (*V*_on_) of 3.8, 3.5, 3.5, and 3.5 V for **TNPhB**, **DNPhCzB**, **NPhDCzB**, and **TCzB**, respectively (Figure 4c,d). Clearly, the asymmetric molecules of **DNPhCzB** and **NPhDCzB** have higher performance than the symmetric **TNPhB** and **TCzB**, showing EQE enhancements up to 18.5% and lower *V*_on_ (**Table** **2**). These performance is among the best results of solution‐processed blue PhOLEDs^17^, ^31^, ^32^, ^33^ and even comparable to that of vacuum‐deposited devices with more complicated device structures (Table S3, Supporting Information).^34^

**Table 2:** Device performances of the solution‐processed blue (**BA–BD**) and white (**WA–WD**) PhOLEDs hosted by the boron‐containing D–A molecules

Device	Hosta)	Guest	*V*_on_ [V]	CE [cd A^−1^]	PE [lm W^−1^]	EQE [%]	CIE
**BA**	**TNPhB**	FIrpic	3.8	31.8	20.0	15.6	(0.21, 0.34)
**BB**	**DNPhCzB** b)	FIrpic	3.5	38.9	30.5	18.4	(0.20, 0.35)
**BC**	**NPhDCzB** c)	FIrpic	3.5	38.5	30.2	18.5	(0.21, 0.34)
**BD**	**TCzB** c)	FIrpic	3.5	32.8	25.8	15.8	(0.22, 0.34)
**WA**	**TNPhB**	FIrpic, Ir(bt)_2_acac	6.7	14.2	5.1	5.8	(0.44, 0.43)
**WB**	**DNPhCzB**	FIrpic, Ir(bt)_2_acac	5.1	27.9	12.5	10.7	(0.41, 0.44)
**WC**	**NPhDCzB**	FIrpic, Ir(bt)_2_acac	5.6	37.0	19.4	14.5	(0.41, 0.44)
**WD**	**TCzB**	FIrpic, Ir(bt)_2_acac	4.6	28.7	15.0	11.0	(0.41, 0.44)

^a)^ With 20 wt% TAPC

^b)^ With 10 wt% TAPC

^c)^ With 30 wt% TAPC.

**Figure 5:**
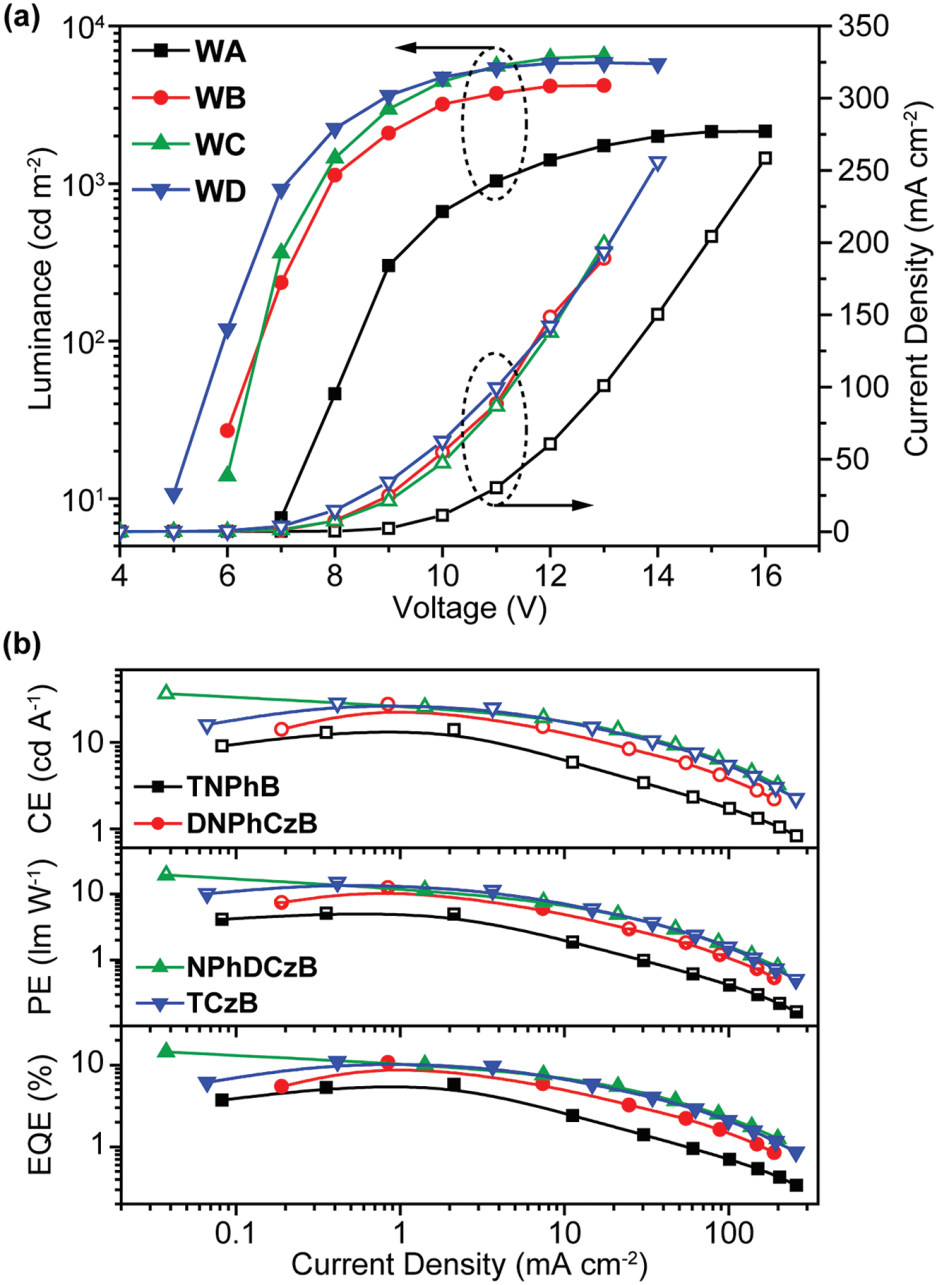
a) Current density–voltage (open symbols) and luminance–voltage (solid symbols) curves, and b) efficiency–current density curves of the white PhOLEDs (**WA**–**WD**) hosted by **TNPhB**, **DNPhCzB**, **NPhDCzB**, and **TCzB** with 20 wt% TAPC, respectively.

The excellent device performance of the solution‐processed FIrpic‐based blue PhOLEDs inspired us to further investigate the application of the boron‐containing host materials on white PhOLEDs with single emission layer (EML). Complementary‐color white‐emitting PhOLEDs (device **WA–WD**) were fabricated with the configuration of ITO/PEDOT: PSS (80 nm)/EML (35 nm)/TmPyPB (60 nm)/Liq (1 nm)/Al (Figure S15, Supporting Information).^11^ The EMLs of **WA–WD** consist of **TNPhB** (**WA**), **DNPhCzB** (**WB**), **NPhDCzB** (**WC**), or **TCzB** (**WD**): 20 wt% TAPC as the host and FIrpic (15 wt%), bis(2‐phenylbenzothiazole) (acetylacetonate)‐iridium(III) (Ir(bt)_2_acac) (1 wt%) as the blue and orange‐red emitter, respectively.^35^ The electroluminescent spectra of **WA–WD** (Figure S16, Supporting Information) show two emission bands for complementary white‐emitting: the blue component peaked at 471 nm with a shoulder at 491 nm is attributed to the electroluminescence of FIrpic, and the other yellow one peaked at 558 nm with a shoulder at 599 nm is due to the emission of Ir(bt)_2_(acac). Importantly, negligible color shifts in these white OLEDs (WOLEDs) can be observed, suggesting the high potential of the materials and device designs for lighting (Figure S16, Supporting Information). The maximum CEs of the white‐emitting PhOLEDs (**WA–WD**) are 14.2, 27.9, 37.0, and 28.7 cd A^−1^; their maximum PEs reach 5.1, 12.5, 19.4, and 15.0 lm W^−1^; and, their maximum EQEs are 5.8%, 10.7%, 14.5%, and 11.0%, respectively (**Figure** **5**a,b). Again, better device performances were observed in the asymmetric host molecules of **DNPhCzB** and **NPhDCzB**.

## 3 Conclusion

In summary, we have developed a series of novel star‐shaped boron‐containing D–A molecules based on an electron‐withdrawing triarylborane core and three peripherial electron‐donating branches of carbazole and diphenylamine in symmetric or asymmetric molecular architecture to address the intrinsic dilemma of boron‐containing compounds in the improvements of stability and solubility. With the combined effects of star‐shaped and asymmetric molecular design strategies, considerably good solubility, high stability, amorphous morphology, bipolar transporting characteristic, and high triplet energy were realized in these star‐shaped and symmetry‐breaking borane derivatives. Consequently, with these facilely prepared molecules as host materials, high‐performance solution‐processed blue and white PhOLEDs were fabricated successfully, exhibiting maximum CEs up to 38.9 and 37.0 cd A^−1^, PEs up to 30.5 and 19.4 lm W^−1^, and EQEs up to 18.5% and 14.5%, respectively. Our strategy, which overcomes the intrinsic difficulties of borane derivatives in solution processing for the first time, demonstrates the great potential of the star‐shaped asymmetric boron‐containing D–A molecules in solution‐processed organic optoelectronic devices.

## Conflict of Interest

The authors declare no conflict of interest.

## Supporting information

Supplementary

## References

[1] D. B. Diaz , A. K. Yudin , Nat. Chem. 2017, 9, 731. 28754930 10.1038/nchem.2814

[2] L. Ji , S. Griesbeck , T. B. Marder , Chem. Sci. 2017, 8, 846. 28572897 10.1039/c6sc04245g PMC5452272

[3] R. Misra , J. Phys. Chem. C﻿﻿ 2017, 121, 5731.

[4] F. Cheng , E. M. Bonder , F. Jäkle , J. Am. Chem. Soc. 2013, 135, 17286. 24191668 10.1021/ja409525j

[5] D. Li , Y. Yuan , H. Bi , D. Yao , X. Zhao , W. Tian , Y. Wang , H. Zhang , Inorg. Chem. 2011, 50, 4825. 21557569 10.1021/ic102554j

[6] X. Long , Y. Gao , H. Tian , C. Dou , D. Yan , Y. Geng , J. Liu , L. Wang , Chem. Commun. 2017, 53, 1649. 10.1039/c6cc09684k 28098269

[7] H. Gao , D. Xu , X. Liu , A. Han , L. Zhou , C. Zhang , Z. Li , J. Dang , Dyes Pigm. 2017, 139, 157.

[8] K. Suzuki , S. Kubo , K. Shizu , T. Fukushima , A. Wakamiya , Y. Murata , C. Adachi , H. Kaji , Angew. Chem., Int. Ed. 2015, 54, 15231. 10.1002/anie.201508270 26563845

[9] M. Numata , T. Yasuda , C. Adachi , Chem. Commun. 2015, 51, 9443. 10.1039/c5cc00307e 25959457

[10] Y. Liu , G. Xie , K. Wu , Z. Luo , T. Zhou , X. Zeng , J. Yu , S. Gong , C. Yang , J. Mater. Chem. C﻿﻿ 2016, 4, 4402.

[11] Y. Tao , X. Guo , L. Hao , R. Chen , H. Li , Y. Chen , X. Zhang , W. Lai , W. Huang , Adv. Mater. 2015, 27, 6939. 26421529 10.1002/adma.201503108

[12] Y. Tao , C. Yang , J. Qin , Chem. Soc. Rev. 2011, 40, 2943. 21369622 10.1039/c0cs00160k

[13] Y. Tao , L. Xu , Z. Zhang , R. Chen , H. Li , H. Xu , C. Zheng , W. Huang , J. Am. Chem. Soc. 2016, 138, 9655. 27403886 10.1021/jacs.6b05042

[14] M. Lin , L. Chi , H. Chang , Y. Huang , K. Tien , C. Chen , C. Chang , C. Wu , A. Chaskar , S. Chou , H. Ting , K. Wong , Y. Liu , Y. Chi , J. Mater. Chem. 2012, 22, 870.

[15] M. Xue , C. Huang , Y. Yuan , L. Cui , Y. Li , B. Wang , Z. Jiang , M. Fung , L. Liao , ACS Appl. Mater. Interfaces 2016, 8, 20230. 27438586 10.1021/acsami.6b05064

[16] Q. Sun , L. Cui , Y. Xie , J. Liang , Z. Jiang , L. Liao , M. Fung , Org. Electron. 2017, 48, 112.

[17] K. S. Yook , J. Y. Lee , Adv. Mater. 2014, 26, 4218. 24807691 10.1002/adma.201306266

[18] S. Ho , S. Liu , Y. Chen , F. So , J. Photonics Energy 2015, 5, 057611.

[19] L. Chen , C. Zhang , G. Lin , H. Nie , W. Luo , Z. Zhuang , S. Ding , R. Hu , S. Su , F. Huang , A. Qin , Z. Zhao , B. Z. Tang , J. Mater. Chem. C﻿﻿ 2016, 4, 2775.

[20] Z. An , R. Chen , J. Yin , G. Xie , H. Shi , T. Tsuboi , W. Huang , Chem. ‐ Eur. J. 2011, 17, 10871. 21887832 10.1002/chem.201101118

[21] H. Li , Y. Wang , K. Yuan , Y. Tao , R. Chen , C. Zheng , X. Zhou , J. Li , W. Huang , Chem. Commun. 2014, 50, 15760. 10.1039/c4cc06636g 25370829

[22] C. Gu , N. Huang , Y. Chen , H. Zhang , S. Zhang , F. Li , Y. Ma , D. Jiang , Angew. Chem., Int. Ed. 2016, 55, 3049. 10.1002/anie.201510723 26822287

[23] H. Li , Y. Tao , R. Chen , G. Xie , C. Zheng , W. Huang , J. Mater. Chem. C﻿﻿ 2017, 5, 4442.

[24] R. Stahl , C. Lambert , C. Kaiser , R. Wortmann , R. Jakober , Chem. ‐ Eur. J. 2006, 12, 2358. 16358349 10.1002/chem.200500948

[25] U. Megerle , F. Selmaier , C. Lambert , E. Riedle , S. Lochbrunner , Phys. Chem. Chem. Phys. 2008, 10, 6245. 18936848 10.1039/b806131a

[26] H. Liu , Q. Bai , L. Yao , D. Hu , X. Tang , F. Shen , H. Zhang , Y. Gao , P. Lu , B. Yang , Y. Ma , Adv. Funct. Mater. 2014, 24, 5881.

[27] Y. Tao , J. Xiao , C. Zheng , Z. Zhang , M. Yan , R. Chen , X. Zhou , H. Li , Z. An , Z. Wang , H. Xu , W. Huang , Angew. Chem. 2013, 125, 10685. 10.1002/anie.201304540 23946117

[28] W. Li , J. Li , D. Liu , Q. Jin , ACS Appl. Mater. Interfaces 2016, 8, 22382. 27517473 10.1021/acsami.6b05355

[29] S. Yang , M. Jiang , Chem. Phys. Lett. 2009, 484, 54.

[30] R. J. Holmes , S. R. Forrest , Y. J. Tung , R. C. Kwong , J. J. Brown , S. Garon , M. E. Thompson , Appl. Phys. Lett. 2003, 82, 2422.

[31] C. W. Lee , J. Y. Lee , Adv. Mater. 2013, 25, 596. 23136111 10.1002/adma.201203180

[32] D. Yu , F. Zhao , C. Han , H. Xu , J. Li , Z. Zhang , Z. Deng , D. Ma , P. Yan , Adv. Mater. 2012, 24, 509. 22213078 10.1002/adma.201104214

[33] G. Sarada , W. Cho , A. Maheshwaran , V. G. Sree , H. Park , Y. Gal , M. Song , S. Jin , Adv. Funct. Mater. 2017, 27, 1701002.

[34] X. Li , J. Zhang , Z. Zhao , L. Wang , H. Yang , Q. Chang , N. Jiang , Z. Liu , Z. Bian , W. Liu , Z. Lu , C. Huang , Adv. Mater. 2018, 30, 1705005. 10.1002/adma.201705005 29380904

[35] T. H. Han , M. R. Choi , C. W. Jeon , Y. H. Kim , S. K. Kwon , T. W. Lee , Sci. Adv. 2016, 2, e1601428. 27819053 10.1126/sciadv.1601428 PMC5091357

